# Inadequate Nutrition Coverage in Outpatient Cancer Centers: Results of a National Survey

**DOI:** 10.1155/2019/7462940

**Published:** 2019-11-22

**Authors:** Elaine B. Trujillo, Katrina Claghorn, Suzanne W. Dixon, Emily B. Hill, Ashlea Braun, Elizabeth Lipinski, Mary E. Platek, Maxwell T. Vergo, Colleen Spees

**Affiliations:** ^1^Division of Cancer Prevention, National Cancer Institute, National Institutes of Health, Rockville, MD 20850, USA; ^2^Abramson Cancer Center, University of Pennsylvania, Philadelphia, PA 19104, USA; ^3^Cambia Health Solutions, Portland, OR, USA; ^4^School of Health and Rehabilitation Sciences, The Ohio State University, Columbus, OH 43210, USA; ^5^Department of Cancer Prevention and Control and Radiation Medicine, Roswell Park Comprehensive Cancer Center and School of Health Related Professions, D'Youville College, Buffalo, NY 14263, USA; ^6^Geisel School of Medicine at Dartmouth, Hanover, NH 03755, USA; ^7^Comprehensive Cancer Center, The Ohio State University, Columbus, OH 43210, USA

## Abstract

Cancer-related malnutrition is associated with poor health outcomes, including decreased tolerance to cancer therapy, greater treatment toxicities, and increased mortality. Medical nutrition therapy (MNT) optimizes clinical outcomes, yet registered dietitian nutritionists (RDNs), the healthcare professionals specifically trained in MNT, are not routinely employed in outpatient cancer centers where over 90% of all cancer patients are treated. The objective of this study was to evaluate RDN staffing patterns, nutrition services provided in ambulatory oncology settings, malnutrition screening practices, and referral and reimbursement practices across the nation in outpatient cancer centers. An online questionnaire was developed by the Oncology Nutrition Dietetic Practice Group (ON DPG) of the Academy of Nutrition and Dietetics and distributed via the ON DPG electronic mailing list. Complete data were summarized for 215 cancer centers. The mean RDN full-time equivalent (FTE) for all centers was 1.7 ± 2.0. After stratifying by type of center, National Cancer Institute-Designated Cancer Centers (NCI CCs) employed a mean of 3.1 ± 3.0 RDN FTEs compared to 1.3 ± 1.4 amongst non-NCI CCs. The RDN-to-patient ratio, based on reported analytic cases, was 1 : 2,308. Per day, RDNs evaluated and counseled an average of 7.4 ± 4.3 oncology patients. Approximately half (53.1%) of the centers screened for malnutrition, and 64.9% of these facilities used a validated malnutrition screening tool. The majority (76.8%) of centers do not bill for nutrition services. This is the first national study to evaluate RDN staffing patterns, provider-to-patient ratios, and reimbursement practices in outpatient cancer centers. These data indicate there is a significant gap in RDN access for oncology patients in need of nutritional care.

## 1. Introduction

The connection of poor and deteriorating nutritional status with adverse clinical outcomes during cancer treatment is well documented. Malnutrition is estimated to occur in up to 80% of cancer patients at some point during or after treatment [1, 2]. Unlike nonwasting malnutrition, cancer-related malnutrition results in accelerated weight loss provoked by systemic inflammation and catabolic factors [3]. This concomitant negative energy balance and skeletal muscle loss is further driven by suboptimal dietary intake and metabolic alterations, including elevated resting energy expenditure, insulin resistance, lipolysis, and proteolysis.

A landmark study in 1980 introduced the concept that significant weight loss may compromise cancer patient survival, independent of conventional prognostic indicators [4]. Since then, the association between weight loss and poor cancer outcomes has been documented in multiple studies [5–12]. According to the Evidence Analysis Library (EAL) of the Academy of Nutrition and Dietetics, there is strong evidence (Grade 1) demonstrating the association between poor nutritional status in adult oncology patients and decreased tolerance to radiation treatment; decreased tolerance to chemotherapy treatment; increased hospital length of stay (LOS); lower quality of life (QoL); and mortality [13].

This downward trajectory can be altered by timely and appropriate nutritional interventions. Medical nutrition therapy (MNT), provided by RDNs, includes individualized nutrition diagnostics, therapies, and counseling aimed at disease management. MNT for oncology patients improves treatment tolerance, reduces treatment interruptions, decreases weight and lean body mass loss, increases quality of life (QoL), decreases unplanned hospitalizations, reduces length of hospital stay, and may improve survival [14–28]. Despite high malnutrition rates in certain cancer patient populations, such as those receiving radiotherapy to the head and neck or esophagus [3], fewer than 60% of those classified as malnourished receive nutritional interventions of any type [29].

Approximately 90% of cancer treatments and care are currently delivered in outpatient clinics [30, 31]. The ambulatory care delivery model has had a negative impact on patient access to RDNs. Current Joint Commission on Accreditation of Healthcare Organizations guidelines mandate nutritional and functional screenings be performed when warranted by the patient's needs or condition and when applicable for the patient's condition in the inpatient setting. These screenings must be completed within 24 hours after admission [32]. In contrast, ambulatory nutritional care standards are ambiguous and inconsistently applied across health care settings, and MNT is not consistently included in multidisciplinary outpatient cancer care [33].

Despite the growing recognition of the importance of providing nutritional care to optimize oncology treatment outcomes and maximize patient QoL, the United States fails to recognize and adequately reimburse MNT as a core component of the multimodal oncology care treatment plan. Conversely, the European Society of Parenteral and Enteral Nutrition [3], the National Institute for Health and Care Excellence of Great Britain [34], and the Victorian Department of Health in Australia [35] all recognize nutrition services as an essential component of cancer care. These organizations call for formalized nutritional screening and assessment, nutrition care plans, and early nutritional intervention when deficits are detected.

A 2016 National Academy of Sciences, Engineering and Medicine workshop, *Examining Access to Nutrition Care in Outpatient Cancer Centers* [36], examined challenges to accessing nutritional care in ambulatory oncology settings. Lack of integration of nutrition services into the cancer health care system and inadequate RDN staffing in cancer centers were identified as major limitations to adequately accessing and implementing oncology nutritional care.

Benchmarking data on access to nutritional care are limited. It is estimated that RDNs provide 0.2 full-time equivalents (FTEs) for ambulatory chemotherapy and radiation clinics [37]. Until clear benchmarks for RDN staffing patterns are established and implemented, the benefits of RDN-delivered oncology MNT will remain unrealized [3, 17]. We conducted a survey to evaluate staffing patterns of oncology RDNs in outpatient cancer centers employing RDNs. Secondary objectives were to determine RDN workload, malnutrition screening practices, and billing patterns.

## 2. Materials and Methods

### 2.1. Participants

Outpatient oncology RDNs in the United States were recruited through the Academy of Nutrition and Dietetics Oncology Nutrition Dietetic Practice Group (ON DPG). An online survey link was distributed via the ON DPG's Electronic Mailing List of approximately 1,000 members. The survey was anonymous; however, the cancer center name was required to avoid cancer center duplication. Recruitment reminders were included in bimonthly ON DPG e-mails. Eligibility criteria included working as an RDN (full time or part time) in an outpatient cancer center; no criteria were defined for outpatient cancer centers. Study procedures were approved by the Ohio State University Institutional Review Board, and informed consent was obtained from all participants prior to participation.

### 2.2. Study Design

A survey was designed by members of the ON DPG Executive Committee and approved by the Academy of Nutrition and Dietetics for distribution using a secure web-based data collection tool (Qualtrics, Provo, UT). The survey was initiated in December 2017, and data collection remained open through July 2018. Respondents had the opportunity to contact ON DPG members affiliated with the research project for clarity as needed.

The online survey consisted of 18 questions. Question responses consisted of multiple choice, ranking, and short answer. A variety of quantitative and qualitative data were collected to provide a rich data set, including cancer center-specific information (e.g., center name and location), oncology patient case load, number of RDN full-time equivalents (FTEs) based on a 40-hour work week employed at each center, RDN nutritional practices and procedures, provision of non-RDN-based nutrition services, availability of electronic technologies for enhancing patient care, malnutrition screening frequency and tools utilized, types of oncology patients typically receiving MNT, referral pathways, and reimbursement mechanisms.

Respondents were prompted to report the number of new analytic cases seen at their cancer center in the most recent complete year. New analytic cases were identified according to criteria defined by the American College of Surgeons' Commission on Cancer as “cases for which the hospital provided the initial diagnosis of cancer and/or for which the hospital contributed to first course of treatment, if those cancers were diagnosed on or after the hospital's reference date and are diseases the Commission on Cancer requires to be abstracted” [38]. Respondents were encouraged to contact their tumor registry for these data or our research team for guidance in retrieving accurate analytic case numbers.

### 2.3. Statistical Analysis

Survey responses were downloaded to a secure server for data analyses. Data from surveys with >50% of questions answered were included in the final data set. Duplicate entries from the same centers were eliminated unless survey answers were missing in which case answers from duplicate entries were pooled to meet the >50% survey answer completion criteria. Descriptive statistics were used to summarize all variables with complete data. Rank frequencies were calculated to identify the most prevalent responses. Additional analyses were conducted to compare National Cancer Institute-Designated Cancer Centers (NCI CCs) versus non-NCI CCs. To derive RDN-to-patient ratio, the analytic patient cases were divided by RDN FTEs individually for each center that provided both data points. Data were normally distributed, and the means were calculated. In addition, the ratios were subanalyzed by NCI CC designation. Qualitative survey responses were reviewed for common themes and selection of illustrative statements. Data were analyzed using SPSS version 25 (IBM SPSS Statistics, Armonk, NY) and a commercially available data visualization software (Tableau, Seattle, WA).

## 3. Results

Of the approximate 1,000 members subscribed to the ON DPG Electronic Mailing List, 310 unique respondents initiated the survey for an overall response rate of about 30%. Of the 310 unique respondents who initiated the survey, 247 had >50% completion and 91 had ≤50% completion. There were duplicates from 15 centers and a quadruplicate from one center, resulting in exclusion of 18 respondents. Partial responses from 14 centers were combined to yield complete data, and 63 incomplete responses were excluded. Approximately 69.4% (*n* = 215) of the surveys met the inclusion criteria and were included in the final analysis (Supplementary [Supplementary-material supplementary-material-1]). As each unique respondent represents one cancer center, the terms respondents and centers will herein be used interchangeably.

**Figure 1:**
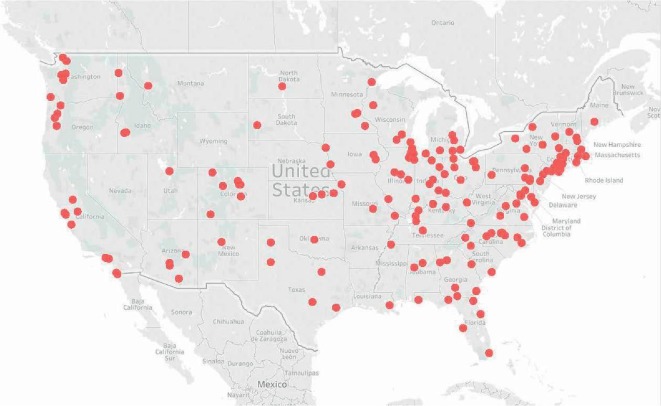
Map of respondents from outpatient cancer centers across the US. The map depicts the location of the 215 cancer centers providing complete data for use in analyses. Forty-three states were represented, including centers from all geographic regions of the continental United States.

Figure 1 illustrates the geographic regions represented by all participating centers. Responders from 43 states completed the survey, representing all major geographic regions of the continental United States. California and Illinois had the highest number of centers represented with 14 responding from these two states. Out of the 215 respondents, 42 were NCI CCs, representing 66.7% of all NCI CCs.

Of the 177 centers who reported an employed dedicated outpatient RDN (either part time or full time), the average length of employment was 10.4 ± 8.2 years (Table 1). A total of 125 centers provided data on both analytic cases as well as RDN FTEs. When stratified by NCI CC designation, NCI CCs reported employment of an RDN for an average of 14.2 ± 8.2 years versus 9.3 ± 7.9 amongst non-NCI CCs. Overall, 1.7 ± 2.0 RDN FTEs were employed in each outpatient oncology center (range: 0–16.6), with FTEs varying by type of service and by NCI CC designation. There was an average of 1.0 ± 1.5 RDN FTEs in Medical Oncology, 0.6 ± 0.6 in Radiation Oncology, and 0.3 ± 0.5 in Infusion. An average of 3.1 ± 3.0 RDN FTEs were reported for NCI CCs versus 1.3 ± 1.4 amongst non-NCI CCs. The mean annual analytic patient cases reported for all centers were 2,073 (range: 0–9351), with head and neck, gastrointestinal, and lung cancer patients receiving the most reported RDN consults. The mean RDN-to-patient ratio was 1 : 2,308, ranging from 1 : 0 to 1 : 53,100.

During an eight-hour work day, RDNs evaluated and/or counseled (defined as initial consults, phone and e-mail consults, and/or one-on-one follow-ups) an average of 7.4 ± 4.3 patients per day (Table 2). When ranked by professional time allocated per week, direct patient care was reported as the activity to which the most time was allocated by RDNs, followed by administrative/non-patient-related activities (e.g., charting, committees, meetings, material development, and public relations) and precepting dietetic interns. When evaluating utilization of internet-based services, RDNs reported frequent use of these services for patient education, scheduling RDN appointments, and classes/webinars.

**Figure 2:**
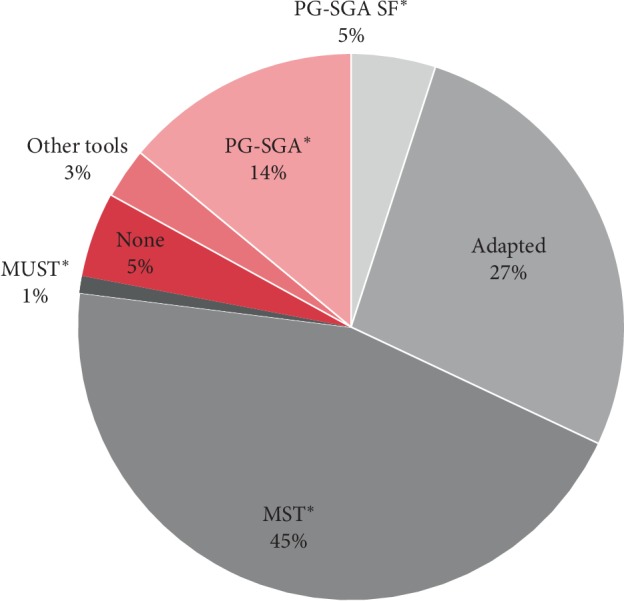
Malnutrition screening tools utilized. The pie graph represents malnutrition screening tools utilized by those routinely screening for malnutrition (*n* = 74). Approximately sixty-five percent of centers who routinely screened for malnutrition used a validated tool (*n* = 48). These validated screening tools are denoted above with an asterisk. PG-SGA SF (Patient-Generated Subjective Global Assessment Short Form); adapted (screening tool that is adapted from its validated version); MST (Malnutrition Screening Tool); MUST (Malnutrition Universal Screening Tool); no screening (no screening tool utilized); other tools (tools not listed above); PG-SGA (Patient-Generated Subjective Global Assessment).

Fifty-three percent of all centers consistently screened for malnutrition (Table 3). When analyzed by NCI CC designation, 45.2% of NCI CCs reported consistently screening, while 55.4% non-NCI CCs consistently screened. Amongst the centers that consistently screened for malnutrition, the majority (64.9%) used a validated screening tool, such as the Malnutrition Screening Tool (MST), the Patient-Generated Subjective Global Assessment (PG-SGA), the PG-SGA Short Form (PG-SGA SF), or the Malnutrition Universal Screening Tool (MUST) (Figure 2). Among those who did not consistently screen for malnutrition, the most commonly reported barriers included lack of referral processes, little to no administrative support, and limited time.

The most frequently utilized mechanism for cancer patient referrals was sporadic identification by clinic or infusion staff (physicians, nurses, nurse practitioners, and/or medical assistants). Other mechanisms included routine screening by RDNs using direct chart reviews followed by patient self-generated subjective assessment form, electronic screening based on questions in the electronic medical record, and other methods (including referrals from other providers, staff meetings, and automatic/routine referrals per center policy). Approximately 76.8% (*n* = 151 responses) of centers reported not billing for nutrition services. Reimbursement results were similar for both NCI CCs (72.7%) and non-NCI CCs (78.0%).

## 4. Discussion

Cancer patients face immense challenges accompanying a cancer diagnosis. Individualized MNT counseling improves QoL, physical functioning, and recovery and reduces symptom severity as compared with non-RDN interventions [39–41]. The demand for evidence-based nutrition information among cancer patients remains high with 30% to 66% of patients reporting that their nutrition information needs were unmet [42]. Despite this, most cancer patients never receive nutritional counseling during their treatment course [3]. Although a growing number of studies document improved outcomes for patients exposed to nutritional interventions led by RDNs, there remains no consensus on staffing patterns nor consistent recommendations mandating malnutrition screening and risk assessment in outpatient oncology clinics [28, 41].

RDN-led interventions resulting in improved QoL and nutrition outcomes include 8 to 9 counseling sessions over a 4.5-month period [16, 28, 41]. Based upon data from this study, the average RDN, counseling 7.5 patients per day, could see 1,013 visits in a 4.5-month timeframe. A desirable RDN-to-patient ratio to achieve this goal would be estimated to be approximately 1 : 120, much less than the current ratio of one RDN to every 2,308 cancer patients. Depending on the stage and type of cancer, unintentional weight loss and malnutrition occur in approximately 30% to 80% of patients. Using a conservative measure of malnutrition risk among cancer patients of 50% based on the literature, we conjecture that over 1,000 oncology patients in our analytic sample would be at risk for malnutrition [4, 43–47]. Based on a mean RDN FTE of 1.7 per center, approximately 600 at risk or possibly malnourished cancer patients could potentially have no access to an RDN.

NCI CCs reported better staffing ratios than non-NCI CCs. Despite this improved staffing, the RDN-to-patient ratio reflects that the RDNs at NCI CCs are responsible for more patients per day than those in the non-NCI CCs. We estimate that if we were able to obtain data from all cancer centers nationwide, the RDN-to-patient ratio would be much more dismal. Centers that do not have RDNs on staff may be utilizing integrative physicians, nurses and nurse practitioners, chiropractors, health education specialists, naturopaths, and/or other providers to provide nutrition services despite the absence of appropriate licensures, nutrition education, and advanced training.

Consistent nutritional screening is a critical first step in the early identification and treatment of patients who are at risk for malnutrition or who are already malnourished. Screening should be used throughout the treatment trajectory and throughout survivorship, as many patients experience various treatment side effects at different times during treatment as well as long-term nutritional consequences of cancer therapies throughout survivorship [48, 49]. Early screening and treatment of malnourished patients in acute care settings reduces hospital length of stay by almost 1.5 days [50, 51]. In our study, approximately half of the cancer centers reported screening for malnutrition. Given the low RDN FTEs and the high patient caseloads, it is not surprising that screening is not consistently practiced. Other than lack of time, common barriers to consistent screening included a lack of standardized referral processes in electronic medical records, and little to no administrative and/or nursing support.

Among the centers that screened for malnutrition, 64.9% used a validated screening tool, including the MST, PG-SGA and PG-SGA SF, and MUST. Despite the availability and the ease of use of these tools, our results indicate that over one-third of practitioners screened using a nonvalidated methodology. The use of nonvalidated nutrition screening tools leads to lack of confidence concerning the accuracy and reliability of the information collected, further limiting the ability to understand the needs of outpatient cancer patients.

Many respondents reported inconsistent patient referral methodologies, such as sporadic referrals by infusion staff. When screening tools are used frequently and consistently, they should trigger a mechanism for automatic patient referrals and complete nutritional assessments. By addressing malnutrition directly, with MNT delivered by RDNs, practitioners can alter the trajectory of weight loss, favorably improving clinical outcomes [14–17, 20, 28].

Reimbursement for nutrition counseling is an obstacle to increasing RDN staffing. Most of the surveyed centers (76.8%) did not bill for nutrition services. Although medical insurance providers are increasingly covering nutrition counseling by RDN practitioners, the Centers for Medicare and Medicaid Services (CMS) do not reimburse nutrition services for oncology patients, but only for nutrition services for diabetes mellitus and renal disease. Although few studies have directly examined the cost-effectiveness of nutritional interventions, there is some evidence supporting an association between oncology nutritional interventions and financial savings related to reduced complications, shorter LOS, and fewer unplanned hospital admissions [37, 52–54]. Identifying and treating malnutrition is essential to maximizing health outcomes and may decrease overall costs of care in cancer centers. Advocacy for financial reimbursement by CMS and other insurers is needed to help cancer centers improve their RDN-to-patient case ratio.

Lastly, the centers that provided online or internet-based services also offered education materials, such as patient scheduling, webinars, and teledietetics, more often than other nutrition services. RDNs utilizing online or internet-based services may be driven by a need to meet their high demand for providing nutrition information to patients, their limited ability to adequately screen and counsel high-risk patients, and a desire to provide evidence-based information to patients.

Limitations to this study include the representativeness of the sample. Although we applied self-directed sampling to intentionally solicit RDNs employed in outpatient settings, we may have excluded those who work in outpatient centers not represented by the listserv used. Additionally, with an estimated number of approximately 1,800 outpatient cancer centers in the US, we acknowledge this survey captured only a subset of cancer centers and therefore results may not be generalizable to all outpatient cancer centers throughout the US. It is important to note that the 30% response rate does not reflect center response but individual RDN response with some RDNs not responding because their colleagues from the same institution already responded. The study is, however, the first to gather data on the lack of RDN availability and the lack of nutrition care services in outpatient cancer centers in the US.

## 5. Conclusions

This study confirms that among the outpatient cancer centers included in this study, many cancer patients treated at these facilities may not have adequate access to RDNs/nutrition care services. The low RDN staffing rates across the nation may be related to the low reimbursement rates for nutrition services in outpatient cancer centers. These benchmarking data inform critical next steps in outpatient oncology care, including the move towards resource allocation to support RDN services in outpatient cancer centers.

## Figures and Tables

**Table 1:** Outpatient oncology RDN staffing patterns.

RDN staffing	Frequency (%) or mean ± SD^a^	Range

Is there a dedicated RDNs for outpatient oncology services at your facility? (*n* = 188)		
Yes	94.1 (177)	—
No	5.9 (11)	—
How many years has your facility employed dedicated RDNs for outpatient oncology?		
All centers (*n* = 159)	10.4 ± 8.2	0–49
NCI-designated cancer centers (*n* = 35)	14.2 ± 8.2	1–30
Non-NCI-designated cancer centers (*n* = 124)	9.3 ± 7.9	0–49

RDN FTE and analytic cases	Mean ± SD^a^	Range

How many RDN full-time equivalents (FTEs) are currently working in any outpatient oncology service?		
Total for All centers (*n* = 199)	1.7 ± 2.0	0–16.6
Total for NCI-Designated cancer centers (*n* = 41)	3.1 ± 3.0	0.025–16.6
Total for non-NCI-designated cancer centers (*n* = 158)	1.3 ± 1.4	0–9.2
How many RDN full-time equivalents (FTEs) are currently working in each outpatient oncology service?^b^ (*n* = 199)		
Medical oncology (*n* = 184)	1.0 ± 1.5	0–12.6
Radiation oncology (*n* = 171)	0.6 ± 0.6	0–4.0
Infusion (*n* = 86)	0.3 ± 0.5	0–2.7
How many new analytic cases were seen at your center in the most recent year for which data are complete?		
All centers (*n* = 128)	2,073 ± 1,991	0–9,351
NCI-designated cancer centers (*n* = 25)	4,297 ± 2,427	30–9,351
Non-NCI-designated cancer centers (*n* = 103)	1,533 ± 1,429	0–7,200

RDN-to-patient ratios	Ratio	Range

All centers (*n* = 124)	1 : 2,308	1 : 0–1 : 53,100^c^
NCI-designated cancer centers (*n* = 25)	1 : 3,587	1 : 10–1 : 47,960
Non-NCI-designated cancer centers (*n* = 99)	1 : 1,984	1 : 0–1 : 53,100^c^

^a^Due to incomplete data and branching logic, the total number of responses varies by question. The total number of complete responses per question is provided next to each response in-line. Percentages are based upon the number of responses out of the total number answering the question. Means and standard deviations are calculated using only complete responses. ^b^Respondents are allowed to select more than one service, as many centers offer all or a combination of these services. ^c^To derive RDN-to-patient ratio. The analytic patient cases were divided by RDN FTEs individually for each center that provided both data points. One center reported having a RDN but no analytic cases (1 : 0). RDN = Registered Dietician Nutritionist; NCI = National Cancer Institute.

**Table 2:** Patient load and services provided.

RDN daily patient work load	Mean ± SD^a^

What is the average number of patients evaluated or counseled by a single outpatient oncology services RDN during an 8-hour work day? (*n* = 172)	7.4 ± 4.3

Services provided	% (*n*)^a^

What online or internet-based services do you offer regarding nutrition education for patients? (*n* = 161)^b^	
Education materials	47.2 (76)
Patient scheduling	24.2 (39)
Classes/webinars	14.3 (23)
Teledietetics/e-coaching	8.7 (14)
None	47.2 (76)
Are nutrition services being provided by non-RDNs? (*n* = 176)	
Yes	5.7 (10)
No	94.3 (166)

^a^Due to incomplete data and branching logic, the total number of responses varies by question. The total number of complete responses per question is provided next to each question in-line. Percentages are based upon the number of responses out of total number answering the question. Means and standard deviations are calculated using only complete responses. ^b^Respondents are allowed to select more than one option and thus, responses may not add up to 100%. RDN = Registered Dietician Nutritionist.

**Table 3:** Malnutrition screening practices.

Malnutrition screening practices	% (*n*)^a^

Does your facility consistently screen for malnutrition? (*n* = 143)	
Yes	53.1 (76)
No	46.9 (67)
Who completes the initial malnutrition screening for oncology outpatients? (*n* = 72)^b^	
Nurse	58.3 (42)
RDN	25.0 (18)
Medical technician	20.8 (15)
Patient (e.g., based on PG-SGA)	16.7 (12)
Medical doctor/oncologist	6.9 (5)
Other^c^	8.3 (6)
If your facility does not consistently screen for malnutrition, do any of the following interfere with malnutrition screening? (*n* = 64)^b^	
No referral process to nutrition services through electronic medical record	46.9 (30)
Little-to-no administrative support	46.9 (30)
Limited time	45.3 (29)
No identified screening tool	31.3 (20)
Little-to-no nursing support	29.7 (19)
No agreement on the screening tool to use among disciplines	25.0 (16)
Other^d^	26.6 (17)

^a^Due to incomplete data and branching logic, the total number of responses varies by question. The total number of complete responses per question is provided next to each question in-line. Percentages are based upon the number of responses out of the total number answering the question. Means and standard deviations are calculated using only complete responses. ^b^Respondents are allowed to select more than one option, and thus, responses may not add up to 100%. ^c^Other includes CAN or MA, combination of RN and RDN, student volunteers, and patient care navigators. ^d^Other includes inadequate RDN staffing, poor implementation of current screening tools center-wide, unable to reimburse for services.

## Data Availability

The survey data used to support the findings of this study may be released upon application to the Oncology Dietetic Practice of the Academy of Nutrition and Dietetics, Attn: Colleen Spees, who can be contacted at colleen.spees@osumc.edu.

## Supplementary Materials

Supplementary Materials Supplementary Figure 1: flow diagram depicting total number of cancer centers completing Nationwide Outpatient Oncology Nutrition Survey. Of the nearly 1,000 members of the Oncology Nutrition Dietetic Practice Group, 310 initiated the nationwide survey. After combining partial responses and excluding duplicate or incomplete responses, a total of 215 unique cancer centers were included in the final analysis.
