# Supplementary material for: Inadequate Nutrition Coverage in Outpatient Cancer Centers: Results of a National Survey
Source: J Oncol. 2019 Nov 22;2019:7462940. doi: 10.1155/2019/7462940 (PMC6893237; doi:10.1155/2019/7462940)
Supplement: Supplementary Materials — Supplementary Figure 1: flow diagram depicting total number of cancer centers completing Nationwide Outpatient Oncology Nutrition Survey. Of the nearly 1,000 members of the Oncology Nutrition Dietetic Practice Group, 310 initiated the nationwide survey. After combining partial responses and excluding duplicate or incomplete responses, a total of 215 unique cancer centers were included in the final analysis. [file 7462940.f1.docx]

**Supplementary Materials**

**215 complete responses from individual centers**

Combined 28 partial responses from 14 centers

Removed 63 incomplete responses

Removed 18 responses from duplicate centers

91 had ≤50% completion rate

247 had >50% completion rate

310 surveys initiated

Supplementary Figure 1: Flow Diagram Depicting Total Number of Cancer Centers Completing Nationwide Outpatient Oncology Nutrition Survey. Of the nearly 1,000 members of the Oncology Nutrition Dietetic Practice Group, 310 initiated the nationwide survey. After combining partial responses and excluding duplicate or incomplete responses, a total of 215 unique cancer centers were included in the final analysis.
